# Survey of Surface Proteins from the Pathogenic *Mycoplasma hyopneumoniae* Strain 7448 Using a Biotin Cell Surface Labeling Approach

**DOI:** 10.1371/journal.pone.0112596

**Published:** 2014-11-11

**Authors:** Luciano Antonio Reolon, Carolina Lumertz Martello, Irene Silveira Schrank, Henrique Bunselmeyer Ferreira

**Affiliations:** 1 Laboratório de Genômica Estrutural e Funcional, Centro de Biotecnologia, UFRGS, Porto Alegre, RS, Brazil; 2 Laboratório de microrganismos diazotróficos, Centro de Biotecnologia, UFRGS, Porto Alegre, RS, Brazil; 3 Programa de Pós-Graduação em Biologia Celular e Molecular, Centro de Biotecnologia, Universidade Federal do Rio Grande do Sul (UFRGS), Porto Alegre, RS, Brazil; 4 Departamento de Biologia Molecular e Biotecnologia, Instituto de Biociências, UFRGS, RS, Brazil; Henan Agricultural Univerisity, China

## Abstract

The characterization of the repertoire of proteins exposed on the cell surface by *Mycoplasma hyopneumoniae* (*M. hyopneumoniae*), the etiological agent of enzootic pneumonia in pigs, is critical to understand physiological processes associated with bacterial infection capacity, survival and pathogenesis. Previous *in silico* studies predicted that about a third of the genes in the *M. hyopneumoniae* genome code for surface proteins, but so far, just a few of them have experimental confirmation of their expression and surface localization. In this work, *M. hyopneumoniae* surface proteins were labeled in intact cells with biotin, and affinity-captured biotin-labeled proteins were identified by a gel-based liquid chromatography-tandem mass spectrometry approach. A total of 20 gel slices were separately analyzed by mass spectrometry, resulting in 165 protein identifications corresponding to 59 different protein species. The identified surface exposed proteins better defined the set of *M. hyopneumoniae* proteins exposed to the host and added confidence to *in silico* predictions. Several proteins potentially related to pathogenesis, were identified, including known adhesins and also hypothetical proteins with adhesin-like topologies, consisting of a transmembrane helix and a large tail exposed at the cell surface. The results provided a better picture of the *M. hyopneumoniae* cell surface that will help in the understanding of processes important for bacterial pathogenesis. Considering the experimental demonstration of surface exposure, adhesion-like topology predictions and absence of orthologs in the closely related, non-pathogenic species *Mycoplasma flocculare*, several proteins could be proposed as potential targets for the development of drugs, vaccines and/or immunodiagnostic tests for enzootic pneumonia.

## Introduction

Mycoplasmas belong to the class Mollicutes and are among the smallest free-living organisms capable of self-replication. Evolutionarily related to Gram-positive bacteria, mycoplasmas have undergone extensive genome reduction, which led to simplification or loss of some metabolic pathways and structural cell components. They are unable to synthesize peptidoglycans or their precursors, and therefore, present no cell wall. The mycoplasma cell membrane is formed from proteins, phospholipids and cholesterol; cholesterol is an essential nutrient to bacterial growth and is responsible for membrane rigidness and stability [1], [2].

*M. hyopneumoniae* is the etiological agent of enzootic pneumonia (EP), a chronic disease characterized by a dry and non-productive cough, most evident when pigs are roused, retarded growth, and inefficient food conversion [3]. In pigs, *M. hyopneumoniae* is found to be attached to the cilia of the tracheal epithelial cells, causing a reduction in ciliary action [4], [5], and predisposing the swine to infection by other pathogens, such as *Pasteurella multocida* [6] and porcine reproductive and respiratory syndrome virus (PRRSV) [7].

*M. hyopneumoniae* adhesion to the swine tracheal epithelial cells is essential to disease establishment, and the characterization of adhesion-mediating molecules has been the focus of most studies on the bacterial mechanisms of virulence and pathogenesis. Bacterial adhesive capability is related to several proteins, such as the well-described P97 adhesin [8], [9], [10] and adhesin-like proteins, such as P216 [11], P159 [12], P102 [13], P146 [14] and P116 [15]. It has been suggested, however, that several other proteins that so far remain uncharacterized are involved in the *M. hyopneumoniae* cell adhesion process [12].

Membrane (integral or associated) proteins are directly exposed on the cell surface and play key roles in cell adhesion, and evasion and/or modulation of the host immune system, events which are important for environmental, bacterial and host cell interactions [16], [17]. The identification of membrane proteins represents a great challenge, especially due to their mainly hydrophobic nature and selective loss during purification, which especially occurs in the precipitation and solubilization steps.

Several methods have been applied to the experimental identification of mycoplasma membrane proteins, especially methods involving selective solubilization the use of detergents, such as Triton X100 [18], [19] and Triton X114 [20]. These methods yield enriched membrane protein fractions, including lipoproteins, but do not completely avoid contamination with cytosolic and ribosomal proteins [21]. Selective labeling using hydrophilic and membrane-impermeable reagents, such as Sulfo-NHS-Biotin, is an alternative way to reduce this contamination and allow the recovery of a more specific fraction of surface exposed proteins [22].

Here, we describe a proteomic approach, based on intact cell surface labeling and labeled protein purification, coupled to gel-based liquid chromatography-tandem mass spectrometry (GeLC-MS/MS), to identify *M. hyopneumoniae* surface exposed proteins. This strategy allowed to identify surface proteins possibly involved in pathogenesis, including some previously annotated as hypothetical proteins. Moreover, a comparative analysis was carried with the closely related non-pathogenic species *Mycoplasma flocculare* (*M. flocculare*), to verify which of the identified surface proteins are found only in the pathogenic counterpart. The potential of some of the identified *M. hyopneumoniae* surface exposed proteins as novel targets for the development of vaccines, diagnostic tests and therapeutic drugs is discussed.

## Methods

### Bacterial strain and culture conditions

The *Mycoplasma hyopneumoniae* pathogenic strain 7448 was isolated from an infected swine from Lindóia do Sul (Santa Catarina, Brazil), and cultured in Friis medium as previously described [23].

### Biotin labeling and affinity recovery of labeled *M. hyopneumoniae* strain 7448 proteins

A cell pellet from 100 ml of fresh *M. hyopneumoniae* 7448 culture was collected by centrifugation at 3360 x g for 15 min. The pellet was washed three times with cold phosphate-buffered saline (PBS; pH 7.2) and resuspended in the same buffer with the addition of 1 mg of sulfosuccinimidyl biotin (EZ-Link Sulfo-NHS-Biotin; Pierce, USA)/ml, in a final concentration of 2 mM of biotin reagent, according to the manufacturer’s instructions. The labeling reaction was performed as previously described [22] for 30 min at 4°C, and the residual sulfo-NHS-biotin was quenched by adding glycine to a final concentration of 100 mM. To remove all unspecific and inactivated Sulfo-NHS-Biotin, the cell suspension was washed twice with 100 mM glycine (final concentration) in PBS. Cells were then lysed by five rounds of sonication (30 s, 20 kHz, 1 min interval between rounds) and labeled proteins were recovered by affinity chromatography in a Monomeric Avidin Resin (Pierce, USA), with gravity flow, using phosphate-buffered saline (PBS, pH 7.0) + 1% Triton X-100 as an equilibration and wash buffer and the same buffer with the addition of biotin to a final concentration of 2 mM was used to block biotin non-reversible binding sites and to elute the bound biotinylated molecule. All steps were performed in the presence of protease inhibitors (Sigma-Aldrich, USA). Chromatography was monitored by measuring the absorbance at 280 nm. As a control, a cell pellet from 100 ml of another fresh *M. hyopneumoniae* 7448 culture was collected by centrifugation at 3360×g for 15 min, washed three times with cold phosphate-buffered saline (PBS; pH 7.2) and lysed by sonication (as described above) prior to biotin labeling of total proteins. After this, labeled proteins were recovered and treated as described above for protein labeling of intact cells. Crude protein extracts were produced in the same way, excluding biotin labeling steps.

Protein samples were concentrated, and salts and detergents were removed by a chloroform/methanol precipitation step and freeze dried until use. All the above procedures of cell culture, and protein labeling and affinity purification were performed in triplicate.

### Electrophoretic prefractionation followed by liquid chromatography tandem mass spectrometry of *M. hyopneumoniae* biotin-labeled proteins

A gel-based liquid chromatography-tandem mass spectrometry (GeLC-MS/MS) [24] approach, using protein prefractionation by sodium dodecyl sulfate-polyacrylamide gel electrophoresis (SDS-PAGE) and in-gel digestion (IGD), followed by liquid chromatography–tandem mass spectrometry (LC-MS/MS), was used for the identification of biotin-labeled proteins of *M. hyopneumoniae* strain 7448. The workflow for GeLC-MS/MS was performed as follows. Freeze-dried protein samples (obtained as described in section 2.2) were resuspended in PBS (pH 7.0) and protein concentration was determined using the Qubit Protein Assay Kit (Invitrogen, USA), according to the manufacturer’s instructions. Samples corresponding to 15 µg of biotin-labeled proteins in PBS, after addition of urea to a final concentration of 8 M, were fractionated by SDS-PAGE on 4–10% Mini-PROTEAN tetra cell precast gels at 80 mV using Tris-SDS running buffer, and stained with Coomassie Brilliant-Blue G250 (Sigma-Aldrich, USA). For each protein sample resolved by SDS-PAGE the corresponding gel lane was divided into 20 slices of similar size (∼15 mm^2^ in area), which were manually excised from the gel and individually processed as follows. They were initially submitted to three washes with 400 µl of 50% acetonitrile and 50 mM ammonium bicarbonate pH 8.0 for 15 min, followed by one washing step with 400 µl of acetonitrile. Gel slices were then incubated with 200 µl of 10 mM DTT in 50 mM ammonium bicarbonate at room temperature for 60 min for reduction, and proteins were subsequently alkylated by incubation with 50 mM iodoacetamide in 50 mM ammonium bicarbonate for 45 min in the dark at room temperature. Gel slices were dried in a CentriVap centrifuge (Labconco, USA). For IGD, gel slices were covered with a trypsin solution (Promega, USA) (20 µg in 1 mL of ammonium bicarbonate 50 mM) and samples were incubated overnight at 37°C. Extraction of the resulting peptides from gel slices was carried out by two successive 1 h incubations with 50 µl of 50% acetonitrile and trifluoroacetic acid (TFA).

Peptides resulting from IGD were separated in a Nanoease C18 (75 µm ID) capillary column by elution with a water/acetonitrile 0.1% formic acid gradient in a capillary liquid chromatography system (Waters, Milford, US). Liquid chromatography was coupled online with an electrospray ionization (ESI) quadrupole time-of-flight (Q-TOF) Ultima API mass spectrometer (Micromass, UK). Data were acquired in data-dependent mode (DDA), and multiple charged peptide ions (+2 and +3) were automatically mass selected and dissociated in MS/MS experiments. Typical LC and ESI conditions included a flow of 200 nL/min, a nanoflow capillary voltage of 3.5 kV, a block temperature of 100°C, and a cone voltage of 100 V. For each replicate, three independent LC-MS/MS measurements were performed. Mass spectrometry was performed in the Unidade de Química de Proteínas e Espectrometria de Massas (Uniprote-MS), Centro de Biotecnologia, UFRGS (Porto Alegre, Brazil).

Protein identification based on peptide MS/MS data was performed using Mascot software (Matrix Science, UK). All tandem mass spectra were searched against a database generated via an *in silico* digest of all proteins encoded by the *M. hyopneumoniae* 7448 genome with the following search parameters: trypsin was used as the cutting enzyme, mass tolerance for the monoisotopic peptide window was set to ±0.2 Da, the MS/MS tolerance window was set to ±0.2 Da, one missed cleavage was allowed, and carbamidomethyl and oxidized methionine were chosen as variable modifications.

### *In silico* analyses of protein identified by GeLC-MS/MS

Cluster of Orthologous in Genomes (COG) annotations were assigned based on sequence similarity searches of CDS entries from *M. hyopneumoniae* strain 7448 (http://www.ncbi.nlm.nih.gov/genome, NC_007332) against the COG annotated proteins database (http://www.ncbi.nlm.nih.gov/COG) [25]. Lipoprotein (LP) *in silico* prediction was performed using LIPOPREDICT (http://www.lipopredict.cdac.in/) and LIPO CBU (http://services.cbu.uib.no/tools/lipo). Ortholog analysis was performed as previously described [26].

## Results

### GeLC-MS/MS analysis of *M. hyopneumoniae* cell surface labeled proteins

Biotin-labeled *M. hyopneumoniae* strain 7448 proteins were resolved by SDS-PAGE, and proteins from gel slices (Figure S1, lane 2 and 3) were subjected to nanoHPLC-nanoESI-Q-TOF-MS/MS identification. MS/MS analyses were performed in replicates (technical replicates) for each of the three biological samples, with virtually the same results. The GeLC-MS/MS identification of labeled intact cell (LIC) samples, resulted in a total of 165 protein identifications (Table S1), corresponding to 59 different protein species (Table S2), which represent approximately 10% of the total proteins encoded by the genome of *M. hyopneumoniae* strain 7448. In control samples from labeled lysed cells (LLC), 96 different proteins species were identified, including several typical intracellular proteins, such as ribosomal proteins, not identified in samples from intact cells (Table S3).

Considering the repertoire of 59 surface exposed proteins detected by GeLC-MS/MS of labeled intact cell (LIC) samples, 34 were identified from two or more gel slices, including well-known adhesion related proteins, such as P76, P97, P102, P146 and P216, along with 19 (32%) hypothetical proteins. Thirty five of these proteins (58.3%) were previously predicted as surface protein [26]. We also performed an *in silico* prediction of *M. hyopneumoniae* 7448 lipoproteins (LPs) (Table S4), and, from the total of 25 predicted LPs (summing those predicted by LIPOPREDICT and LIPO CBU tools), 16 (64%) were detected in our survey of surface exposed proteins. Among the MS-detected surface proteins predicted *in silico* as such or as LPs, there are well-known adhesion related proteins, such as P76, P97, P102, P146 and P216, 46 K surface antigen precursor, ABC transporter xylose-binding lipoprotein, and prolipoprotein p65, along with 16 hypothetical proteins.

A total of 21 proteins out of the 59 identified as surface exposed by GeLC-MS/MS of labeled intact cell (LIC) samples (35%) were not predicted *in silico* as such [26] or as lipoproteins (our results) (Table S2). This group includes proteins traditionally involved in intracellular processes. An additional analysis of these proteins reveled that at least for some of them, like pyruvate dehydrogenase subunits, glyceraldehyde 3-phosphate dehydrogenase (GAPDH), L-lactate dehydrogenase, elongation factor Tu (Ef-Tu), molecular chaperone DnaK, and NADH-dependent flavin oxidoreductase, there are previous evidences in the literature (see Discussion) suggesting their surface localization in mycoplasmas or other pathogenic bacteria.

### Functional classification of *M. hyopneumoniae* surface proteins from LIC samples identified by GeLC-MS/MS

**Figure 1:**
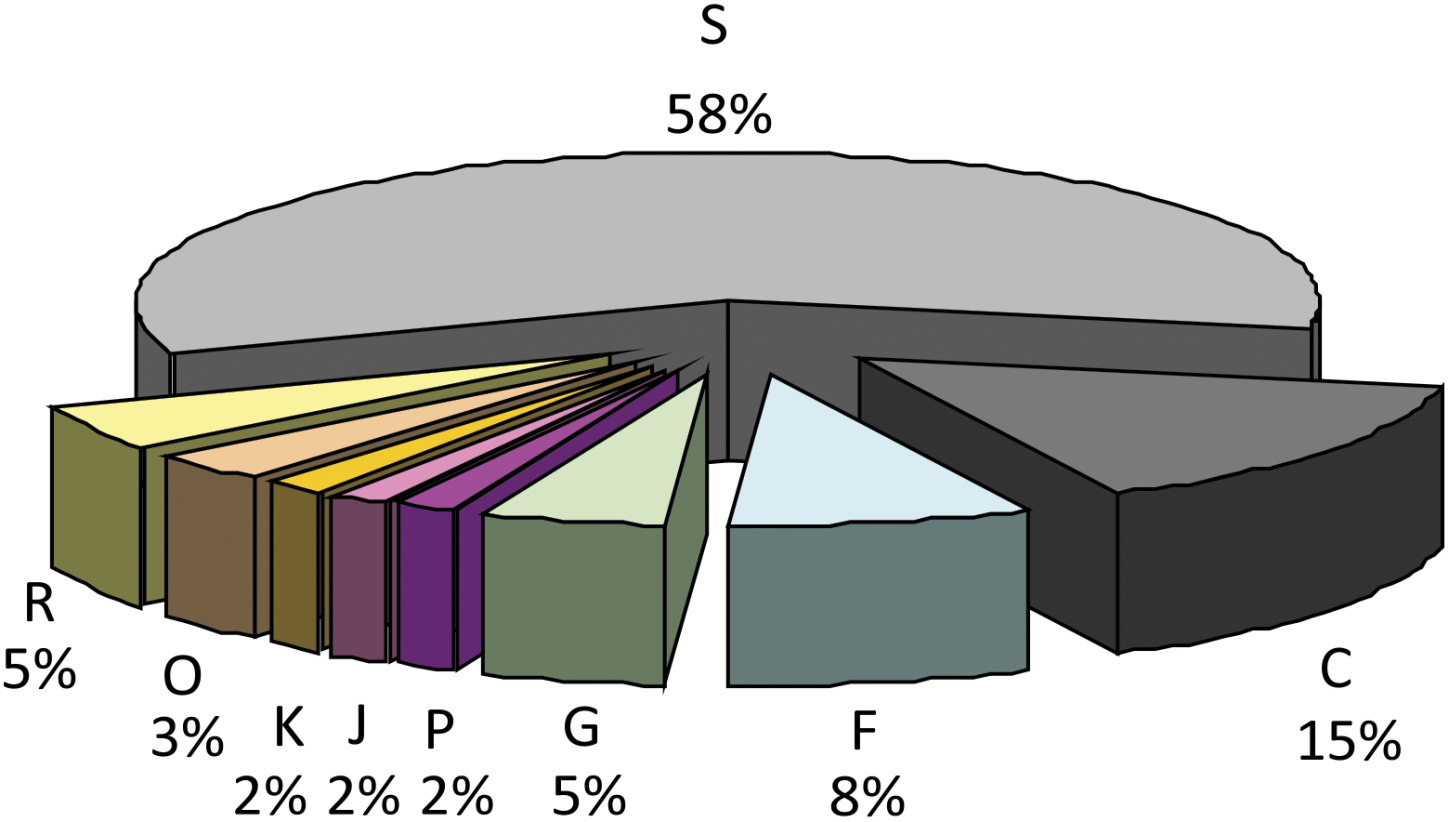
Functional analysis of *M. hyopneumoniae* surface proteins identified by GeLC-MS/MS in LIC samples. Percentages of proteins predicted in each functional category are indicated in the sectors of the circle. COG functional classes are as follows. Major class information storage and processes: (J) Translation, ribosomal structure and biogenesis, (K) Transcription; Major class cellular processes: (O) Post-translational modification, protein turnover, and chaperones, (P) Inorganic ion transport and metabolism; Major class Metabolism: (C) Energy production and conversion, (G) Carbohydrate transport and metabolism, (F) Nucleotide transport and metabolism; Major class poorly characterized: (R) General function prediction only, (S) Function unknown.

In an attempt to infer potential physiological/functional features, the functional classification of the 59 proteins experimentally identified as surface exposed was performed based on the COG [25] (Figure 1, Table S2). According to COG, more than half of the GeLC-MS/MS identified *M. hyopneumoniae* surface proteins (35 out of 59; 58%) from LIC samples were classified as having unknown function, a class which included adhesion-related (6 proteins) and hypothetical proteins (19 proteins). Energy production and conversion was the second well-represented class (9 out of 59; 15%), followed by nucleotide transport and metabolism (5 out of 59; 8%), carbohydrate transport and metabolism (3 out of 59; 5%), general function prediction (3 out of 59; 5%), posttranslational modification/protein turnover/chaperones (2 out of 59; 2%), inorganic ion transport and metabolism (1 out of 59; 1.6%), translation, ribosomal structure and biogenesis (1 out of 59; 1.6%) and translation (1 out of 59; 1.6%).

### Topology predictions of *M. hyopneumoniae* surface proteins from LIC samples identified by GeLC-MS/MS

*In silico* topology predictions were performed for the 59 proteins experimentally identified as surface exposed. These predictions (Table S2) showed that 19 of them had transmembrane domains (TM proteins), including the adhesion related proteins P76, P97, P102, P146 and P216, the transporter potassium uptake protein, and the cell division protein. Interestingly, other 10 of the 19 putative transmembrane proteins also presented adhesion-like predicted topologies, consisting of a transmembrane helix and a large tail exposed at the cell surface. These proteins are protein MHP7448_0372, 46 K surface antigen precursor, periplasmic sugar-binding protein, and 7 hypothetical proteins (MHP7448_0138, MHP7448_0352, MHP7448_0373, MHP7448_0467, MHP7448_0468, MHP7448_0629, and MHP7448_0661).

Considering the experimental demonstration of surface exposure, adhesin-like topology predictions and absence of orthologue in the closely related, non-pathogenic species *M. flocculare*, 12 of the proteins identified by GeLC-MS/MS in LIC samples could be selected as potential targets for the development of drugs, vaccines and/or immunodiagnostic tests (Table 1). These include proteins previously related to pathogenicity in bacteria (NADH-dependent flavin oxidoreductase, and 46 K surface antigen precursor), and several previously uncharacterized *M. hyopneumoniae* hypothetical proteins.

**Table 1:** Adhesin topology prediction and additional criteria for the selection of the identified surface exposed proteins as potential targets for the development of drugs, vaccines and/or immunodiagnostic tests.

Locus Tag^1^	Proteinproduct^2^	Protein name	Adhesin-liketopology^3^	Absence in*M. flocculare* 4	Studies suggested a surfacelocalization^5^
MHP7448_0088	YP_287488.1	Hypothetical protein MHP7448_0088		•	
MHP7448_0138	YP_287535.1	Hypothetical protein MHP7448_0138	•		
MHP7448_0234	YP_287631.1	Periplasmic sugar-binding protein	•	•	[46]
MHP7448_0309	YP_287705.1	NADH-dependent flavin oxidoreductase		•	[48], [49], [50]
MHP7448_0324	YP_287719.1	Hypothetical protein MHP7448_0324		•	
MHP7448_0333	YP_287728.1	Hypothetical protein MHP7448_0333		•	
MHP7448_0352	YP_287746.1	Hypothetical protein MHP7448_0352	•		[29]
MHP7448_0372	YP_287766.1	Protein MHP7448_0372	•		
MHP7448_0373	YP_287767.1	Hypothetical protein MHP7448_0373	•		[30]
MHP7448_0513	YP_287902.1	46 K surface antigen precursor	•		
MHP7448_0629	YP_288014.1	Hypothetical protein MHP7448_0629	•		
MHP7448_0661	YP_288046.1	Hypothetical protein MHP7448_0661	•		

1 Locus tag as defined for *M. hyopneumoniae* strain 7448 (http://www.ncbi.nlm.nih.gov/genome, NC_007332).

2 Protein product according to NCBI database (http://www.ncbi.nlm.nih.gov).

3 Topology prediction similar to well known adhesins (Table S2).

4 No ortholog found closely related, non-pathogenic species *M. flocculare*.

5 Published literature.

## Discussion

Cell surface proteins are directly exposed to the environment and can mediate important pathogen-host interactions, such as adhesion, cell signaling and immune modulation, all of which are relevant for pathogenesis. For *M. hyopneumoniae*, it is predicted that about a third of the genes in its genome code for surface proteins [26], but so far, just a few of them have experimental confirmation of their expression and surface localization. Here, we employed a biotin cell surface labeling approach followed by avidin affinity recovery of labeled proteins coupled to identification by GeLC-MS/MS to survey the exposed surface proteins from *M. hyopneumoniae*. Experimental demonstration of surface exposure, adhesion-like topology predictions and absence of orthologs in the closely related, non-pathogenic *M. flocculare* allowed to propose several proteins as candidates for the development of drugs, vaccines and/or immunodiagnostic tests for enzootic pneumonia.

Biotinylation has already been described as useful for the specific labeling of exposed tails of surface proteins [21], [22]. The GeLC-MS/MS workflow was chosen for the qualitative identification of biotin-labeled *M. hyopneumoniae* surface proteins due to some advantages for hydrophobic protein analysis, including complete solubilization by SDS and removal of detergent and salts [24]. The biotinylation-GeLC-MS/MS combined approach successfully enriched samples and identified *M. hyopneumoniae* surface proteins, with no ribosomal protein recovery and with the identification of few classical cytoplasm proteins (Table S2). Besides, a large proportion of adhesion related proteins and LPs was selectively recovered and identified from LIC samples, in contrast with labeled lysed cell (LLC) control samples (cells lysed prior to labeling), from which proteins involved in a broad range of cellular functions, localized in both cytoplasm and membrane were identified (Table S3).

The adherence of *M. hyopneumoniae* to tracheal host cells is a crucial step for colonization and establishment of disease in infected pigs [1], [3], [16]. Adhesins play key roles in the process of pathogen binding to a host cell, although cell adhesion is a multifactorial process that involves surface proteins from both bacterial and host cells [27]. Well-known *M. hyopneumoniae* adhesins, namely P97, P97-like, P216, P102, and P76 proteins, which have been associated with binding to porcine cilia, heparin, fibronectin and plasminogen [8], [9], [10], [11], [13], had their surface localization confirmed by GeLC-MS/MS identification in LIC samples (Table S2). Other well represented proteins in our proteomic survey were LPs, among which were ABC transporter xylose-binding lipoprotein, and P60-like lipoprotein. ABC transporter xylose-binding lipoprotein is similar to the outer membrane lipoprotein P48 of some *Mycoplasma* species [28], described as an immunomodulatory protein required for intracellular invasion. The P60-like lipoprotein ortholog of *Mycoplasma hominis* was previously described [29] as a surface protein associated with P80 protein, whose *M. hyopneumoniae* ortholog (MHP7448_352) was also identified in our survey.

The GeLC-MS/MS protein identification in LIC samples also revealed the presence of 19 different hypothetical proteins in the *M. hyopneumoniae* cell surface. This corresponds to 6.3% of the total of 298 *M. hyopneumoniae* 7448 CDS products annotated as hypothetical proteins [26]. The products of two of these CDSs (MHP7448_0373 and MHP7448_0662) have been recently associated with cell adhesion. The MHP7448_0373 hypothetical protein was described as a heparin binding protein in porcine cilia [30], and the MHP7448_0662 hypothetical protein, which is a paralog of P102 was described as an adhesion protein able to bind fibronectin extracellular compounds [31] overexpressed in pathogenic strains [32]. Furthermore, our topology analysis showed that five of the 19 identified hypothetical proteins have adhesin-like topologies (Table S2).

Some proteins traditionally related to cellular processes that occur mainly in the cytoplasm were also identified in the *M. hyopneumoniae* cell surface. At least some of them, namely pyruvate dehydrogenase, GAPDH, L-lactate dehydrogenase, Ef-Tu and DnaK, have also been described as bacterial surface components, with involvement in bacterium-host interaction and pathogenicity. Pyruvate dehydrogenase, an immunogenic *M. hyopneumoniae* protein [33], [34], was described as a surface protein involved in the bacterial binding to the host extracellular matrix in *Mycoplasma pneumoniae* [35]. GAPDH, usually located in the cytoplasm and known to play a central role as a glycolytic enzyme, has been identified on the surface of pathogenic bacteria and was related to pathogenic processes [36], such as adhesion and host matrix binding [37], immunomodulation and immune evasion [38]. L-lactate dehydrogenase, also known as P36, was described as immunogenic for pigs infected with *M. hyopneumoniae* [39]. The translational factor Ef-Tu was also identified as an immunogenic cell surface protein in mycoplasmas [18]. The molecular chaperone DnaK has been shown to be surface accessible in *M. hyopneumoniae* [40] and was suggested as a surface or secreted protein of several other pathogenic bacteria, as *Bacillus anthracis* [41] and *Mycobacterium tuberculosis* [42].

The identification of the same protein species from two or more gel slices was taken as further evidence of proteolytic post-translational processing. This was observed for 34 of the 59 different protein species identified by GeLC-MS/MS in LIC samples, including adhesins and hypothetical proteins (Table S1). Proteolytic post-translational processing of *M. hyopneumoniae* proteins has been previously shown [43], [44], and an alternative explanation based on protein degradation during sample processing was discarded in our survey due to the use of broad range protease inhibitors.

Nearly two thirds (39 out of 59) of the GeLC-MS/MS identified *M. hyopneumoniae* 7448 proteins in LIC samples was predicted *in silico* as surface proteins, considering the previous work by Siqueira *et al.* [26] and our complementary LP prediction. The extensive overlap between our proteomic approach and the *in silico* predictions is another indicator of the success of the selective surface biotin labeling, increasing the confidence of our results. Our proteomic results also contributed to better understanding of the *M. hyopneumoniae* 7448 repertoire of surface exposed proteins by also detecting, as discussed above, annotated (16) and hypothetical (5) proteins previously not predicted as bacterial surface components (Table S2). Considering sensitivity limitations of both labeling and MS detection [21], the identified proteins can be considered to be among the more abundant protein species on the *M. hyopneumoniae* 7448 cell surface.

The pathogenic *M. hyopneumoniae* and the non-pathogenic *M. flocculare* cohabit the swine respiratory tract and both species adhere to the cilia of tracheal epithelial cells, but just *M. hyopneumoniae* causes tissue damage [45]. Considering the extensive genetic similarity between *M. hyopneumoniae* and *M. flocculare* [26], we also surveyed the *M. flocculare* genome for orthologs of the *M. hyopneumoniae* 7448 proteins identified in LIC samples. Although *M. hyopneumoniae* 7448 and *M. flocculare* share nearly 90% of their predicted repertoire of surface proteins [26], 5 out of the 59 (8%) proteins identified in LIC samples are not found in *M. flocculare*. The *M. hyopneumoniae* proteins not shared with *M. flocculare* are three hypothetical proteins (MHP7448_0088, MHP7448_0324 and MHP7448_0333); a periplasmic sugar-binding protein that serves as primary receptor for diverse solutes in transport system, chemotaxis and signaling [46]; and a NAD-dependent flavin oxidoreductase. Interestingly, the involvement of oxidoreductase enzymes in pathogenicity has been described [47], [48], [49], and recent studies demonstrated that the *Mycobacterium avium* subsp. *paratuberculosis* NADH- flavin oxidoreductase from is essential for invasion of epithelial cells [50].

Besides the absence in *M. flocculare*, another aspect that calls attention to the periplasmic sugar-binding protein and to some other surface proteins identified in *M. hyopneumoniae* LIC samples was their adhesin-like topology. These criteria, along with previous studies of possible involvement of ortholog proteins in pathogen-host interactions, allowed us to propose at least 12 proteins (Table 1) as novel and promising targets for future studies.

## Conclusion

The continuous development of immunization strategies, diagnosis tests and new drugs and antibiotics for disease treatment is essential for the control and prevention of EP [51]. Besides, some aspects of *M. hyopneumoniae* pathogenicity still need further clarification, including cell adhesion and immunomodulation [2], [5], [13]. Bacterial cell surface proteins play key roles in several cellular events important for bacterial growth, colonization and host inception. The selective labeling of surface proteins and their identification by GeLC-MS/MS described here provided new insights about cell surface protein composition in *M. hyopneumoniae* and allowed to point out novel and promising targets for the development of vaccines, diagnostic tests and therapeutic drugs. Further insights on *M. hyopneumoniae* pathogenicity are expected when the approaches standardized here were coupled to quantitative proteomic strategies for the comparison of the surface protein content of pathogenic and non-pathogenic *M. hyopneumoniae* strains.

## Supporting Information

Figure S1 **Biotin labeling and affinity capture of M. hyopneumoniae proteins from labeled lysed cell (LLC) and labeled intact cell (LIC) samples.** SDS-PAGE 10%, and stained with Coomassie Brilliant Blue. Lane M – marker, Precision Plus prestained protein standards (Bio-Rad). Lane 1– crude protein extracts from M. hyopneumoniae 7448 (15 µg). Lane 2– avidin affinity capture of proteins from M. hyopneumoniae LLC samples (15 µg). Lane 3 - avidin affinity capture of proteins from M. hyopneumoniae LIC samples (15 µg). Bands 1 to 20 were subjected to Nano-LC/MS/MS analysis.
(TIF)

Table S1 ***Proteins identified by GeLC-MS/MS in labeled intact cell (LIC) samples.***
(XLSX)

Table S2 **Different protein species identified by GeLC-MS/MS in labeled intact cell (LIC) samples.**
(XLSX)

Table S3 **Different protein species identified by GeLC-MS/MS in control samples from labeled lysed cells (LLC).**
(XLSX)

Table S4 **Lipoprotein predictions.**
(XLSX)

## References

[1] Fadiel A, Eichenbaum KD, El SN, Epperson B (2007) Mycoplasma genomics: tailoring the genome for minimal life requirements through reductive evolution. Front Biosci 12: 2020–2028. 2207 [pii]. 10.2741/2207 17127440

[2] Razin S, Yogev D, Naot Y (1998) Molecular biology and pathogenicity of mycoplasmas. Microbiol Mol Biol Rev 62: 1094–1156. 9841667 10.1128/mmbr.62.4.1094-1156.1998 PMC98941

[3] Maes D, Verdonck M, Deluyker H, de KA (1996) Enzootic pneumonia in pigs. Vet Q 18: 104–109 10.1080/01652176.1996.9694628 [doi]. 8903144

[4] DeBey MC, Ross RF (1994) Ciliostasis and loss of cilia induced by Mycoplasma hyopneumoniae in porcine tracheal organ cultures. Infect Immun 62: 5312–5318. 7960110 10.1128/iai.62.12.5312-5318.1994 PMC303270

[5] Zielinski GC, Ross RF (1993) Adherence of Mycoplasma hyopneumoniae to porcine ciliated respiratory tract cells. Am J Vet Res 54: 1262–1269. 8214893

[6] Amass SF, Clark LK, van Alstine WG, Bowersock TL, Murphy DA, et al (1994) Interaction of Mycoplasma hyopneumoniae and Pasteurella multocida infections in swine. J Am Vet Med Assoc 204: 102–107. 8125807

[7] Thacker EL, Halbur PG, Ross RF, Thanawongnuwech R, Thacker BJ (1999) Mycoplasma hyopneumoniae potentiation of porcine reproductive and respiratory syndrome virus-induced pneumonia. J Clin Microbiol 37: 620–627. 9986823 10.1128/jcm.37.3.620-627.1999 PMC84495

[8] Zhang Q, Young TF, Ross RF (1994) Microtiter plate adherence assay and receptor analogs for Mycoplasma hyopneumoniae. Infect Immun 62: 1616–1622. 8168922 10.1128/iai.62.5.1616-1622.1994 PMC186367

[9] Djordjevic SP, Cordwell SJ, Djordjevic MA, Wilton J, Minion FC (2004) Proteolytic processing of the Mycoplasma hyopneumoniae cilium adhesin. Infect Immun 72: 2791–2802. 15102789 10.1128/IAI.72.5.2791-2802.2004 PMC387856

[10] Deutscher AT, Jenkins C, Minion FC, Seymour LM, Padula MP, et al (2010) Repeat regions R1 and R2 in the P97 paralogue Mhp271 of Mycoplasma hyopneumoniae bind heparin, fibronectin and porcine cilia. Mol Microbiol 78: 444–458 10.1111/j.1365-2958.2010.07345.x [doi]. 20879998

[11] Wilton J, Jenkins C, Cordwell SJ, Falconer L, Minion FC, et al (2009) Mhp493 (P216) is a proteolytically processed, cilium and heparin binding protein of Mycoplasma hyopneumoniae. Mol Microbiol 71: 566–582 MMI6546 [pii]; 10.1111/j.1365-2958.2008.06546.x [doi]. 19040640

[12] Burnett TA, Dinkla K, Rohde M, Chhatwal GS, Uphoff C, et al (2006) P159 is a proteolytically processed, surface adhesin of Mycoplasma hyopneumoniae: defined domains of P159 bind heparin and promote adherence to eukaryote cells. Mol Microbiol 60: 669–686 MMI5139 [pii]; 10.1111/j.1365-2958.2006.05139.x [doi]. 16629669

[13] Seymour LM, Jenkins C, Deutscher AT, Raymond BB, Padula MP, et al (2012) Mhp182 (P102) binds fibronectin and contributes to the recruitment of plasmin(ogen) to the Mycoplasma hyopneumoniae cell surface. Cell Microbiol 14: 81–94 10.1111/j.1462-5822.2011.01702.x [doi]. 21951786

[14] Bogema DR, Deutscher AT, Woolley LK, Seymour LM, Raymond BB, et al. (2012) Characterization of cleavage events in the multifunctional cilium adhesin Mhp684 (P146) reveals a mechanism by which Mycoplasma hyopneumoniae regulates surface topography. MBio 3. mBio.00282-11 [pii];10.1128/mBio.00282-11 [doi]. 10.1128/mBio.00282-11 PMC3322551 22493032

[15] Seymour LM, Deutscher AT, Jenkins C, Kuit TA, Falconer L, et al (2010) A processed multidomain mycoplasma hyopneumoniae adhesin binds fibronectin, plasminogen, and swine respiratory cilia. J Biol Chem 285: 33971–33978 M110.104463 [pii]; 10.1074/jbc.M110.104463 [doi]. 20813843 PMC2962497

[16] Rottem S (2003) Interaction of mycoplasmas with host cells. Physiol Rev 83: 417–432 10.1152/physrev.00030.2002 [doi]. 12663864

[17] You XX, Zeng YH, Wu YM (2006) Interactions between mycoplasma lipid-associated membrane proteins and the host cells. J Zhejiang Univ Sci B 7: 342–350 10.1631/jzus.2006.B0342 [doi]. 16615163 PMC1462930

[18] Cacciotto C, Addis MF, Pagnozzi D, Chessa B, Coradduzza E, et al (2010) The liposoluble proteome of Mycoplasma agalactiae: an insight into the minimal protein complement of a bacterial membrane. BMC Microbiol 10: 225 1471-2180-10-225 [pii]; 10.1186/1471-2180-10-225 [doi]. 20738845 PMC2941501

[19] Corona L, Cillara G, Tola S (2013) Proteomic approach for identification of immunogenic proteins of Mycoplasma mycoides subsp. capri. Vet Microbiol 167: 434–439 S0378-1135(13)00442-2 [pii]; 10.1016/j.vetmic.2013.08.024 [doi]. 24090811

[20] Krasteva I, Liljander A, Fischer A, Smith DG, Inglis NF, et al (2014) Characterization of the in vitro core surface proteome of Mycoplasma mycoides subsp. mycoides, the causative agent of contagious bovine pleuropneumonia. Vet Microbiol 168: 116–123 S0378-1135(13)00498-7 [pii]; 10.1016/j.vetmic.2013.10.025 [doi]. 24332827

[21] Cordwell SJ (2006) Technologies for bacterial surface proteomics. Curr Opin Microbiol 9: 320–329 S1369-5274(06)00057-9 [pii]; 10.1016/j.mib.2006.04.008 [doi]. 16679049

[22] Cullen PA, Xu X, Matsunaga J, Sanchez Y, Ko AI, et al (2005) Surfaceome of Leptospira spp. Infect Immun 73: 4853–4863 73/8/4853 [pii]; 10.1128/IAI.73.8.4853-4863.2005 [doi]. 16040999 PMC1201201

[23] Vasconcelos AT, Ferreira HB, Bizarro CV, Bonatto SL, Carvalho MO, et al (2005) Swine and poultry pathogens: the complete genome sequences of two strains of Mycoplasma hyopneumoniae and a strain of Mycoplasma synoviae. J Bacteriol 187: 5568–5577 187/16/5568 [pii]; 10.1128/JB.187.16.5568-5577.2005 [doi]. 16077101 PMC1196056

[24] Piersma SR, Warmoes MO, de Wit M, de Reus I, Knol JC, et al (2013) Whole gel processing procedure for GeLC-MS/MS based proteomics. Proteome Sci 11: 17 1477-5956-11-17 [pii]; 10.1186/1477-5956-11-17 [doi]. 23617947 PMC3656797

[25] Tatusov RL, Fedorova ND, Jackson JD, Jacobs AR, Kiryutin B, et al (2003) The COG database: an updated version includes eukaryotes. BMC Bioinformatics 4: 41 10.1186/1471-2105-4-41 [doi];1471-2105-4-41 [pii]. 12969510 PMC222959

[26] Siqueira FM, Thompson CE, Virginio VG, Gonchoroski T, Reolon L, et al (2013) New insights on the biology of swine respiratory tract mycoplasmas from a comparative genome analysis. BMC Genomics 14: 175 1471-2164-14-175 [pii]; 10.1186/1471-2164-14-175 [doi]. 23497205 PMC3610235

[27] Krause DC (1996) Mycoplasma pneumoniae cytadherence: unravelling the tie that binds. Mol Microbiol 20: 247–253. 8733224 10.1111/j.1365-2958.1996.tb02613.x

[28] Hall RE, Kestler DP, Agarwal S, Goldstein KM (1999) Expression of the monocytic differentiation/activation factor P48 in Mycoplasma species. Microb Pathog 27: 145–153 10.1006/mpat.1999.0293 [doi];S0882-4010(99)90293-0 [pii]. 10455005

[29] Hopfe M, Hoffmann R, Henrich B (2004) P80, the HinT interacting membrane protein, is a secreted antigen of Mycoplasma hominis. BMC Microbiol 4: 46 1471-2180-4-46 [pii]; 10.1186/1471-2180-4-46 [doi]. 15579213 PMC539234

[30] Deutscher AT, Tacchi JL, Minion FC, Padula MP, Crossett B, et al (2012) Mycoplasma hyopneumoniae Surface proteins Mhp385 and Mhp384 bind host cilia and glycosaminoglycans and are endoproteolytically processed by proteases that recognize different cleavage motifs. J Proteome Res 11: 1924–1936 10.1021/pr201115v [doi]. 22229926

[31] Bogema DR, Scott NE, Padula MP, Tacchi JL, Raymond BB, et al (2011) Sequence TTKF downward arrow QE defines the site of proteolytic cleavage in Mhp683 protein, a novel glycosaminoglycan and cilium adhesin of Mycoplasma hyopneumoniae. J Biol Chem 286: 41217–41229 M111.226084 [pii]; 10.1074/jbc.M111.226084 [doi]. 21969369 PMC3308835

[32] Pinto PM, Klein CS, Zaha A, Ferreira HB (2009) Comparative proteomic analysis of pathogenic and non-pathogenic strains from the swine pathogen Mycoplasma hyopneumoniae. Proteome Sci 7: 45 1477-5956-7-45 [pii]; 10.1186/1477-5956-7-45 [doi]. 20025764 PMC2804596

[33] Pinto PM, Chemale G, de Castro LA, Costa AP, Kich JD, et al (2007) Proteomic survey of the pathogenic Mycoplasma hyopneumoniae strain 7448 and identification of novel post-translationally modified and antigenic proteins. Vet Microbiol 121: 83–93 S0378-1135(06)00472-X [pii]; 10.1016/j.vetmic.2006.11.018 [doi]. 17182197

[34] Matic JN, Wilton JL, Towers RJ, Scarman AL, Minion FC, et al. (2003) The pyruvate dehydrogenase complex of Mycoplasma hyopneumoniae contains a novel lipoyl domain arrangement. Gene 319: 99–106. S0378111903007984 [pii]. 10.1016/s0378-1119(03)00798-4 14597175

[35] Thomas C, Jacobs E, Dumke R (2013) Characterization of pyruvate dehydrogenase subunit B and enolase as plasminogen-binding proteins in Mycoplasma pneumoniae. Microbiology 159: 352–365 mic.0.061184-0 [pii]; 10.1099/mic.0.061184-0 [doi]. 23197176

[36] Seidler NW (2013) GAPDH, as a virulence factor. Adv Exp Med Biol 985: 149–178 10.1007/978-94-007-4716-6_5 [doi]. 22851449

[37] Alvarez RA, Blaylock MW, Baseman JB (2003) Surface localized glyceraldehyde-3-phosphate dehydrogenase of Mycoplasma genitalium binds mucin. Mol Microbiol 48: 1417–1425. 3518 [pii]. 10.1046/j.1365-2958.2003.03518.x 12787366

[38] Terao Y, Yamaguchi M, Hamada S, Kawabata S (2006) Multifunctional glyceraldehyde-3-phosphate dehydrogenase of Streptococcus pyogenes is essential for evasion from neutrophils. J Biol Chem 281: 14215–14223 M513408200 [pii]; 10.1074/jbc.M513408200 [doi]. 16565520

[39] Frey J, Haldimann A, Kobisch M, Nicolet J (1994) Immune response against the L-lactate dehydrogenase of Mycoplasma hyopneumoniae in enzootic pneumonia of swine. Microb Pathog 17: 313–322 S0882-4010(84)71077-1 [pii]; 10.1006/mpat.1994.1077 [doi]. 7723658

[40] Chou SY, Chung TL, Chen RJ, Ro LH, Tsui PI, et al (1997) Molecular cloning and analysis of a HSP (heat shock protein)-like 42 kDa antigen gene of Mycoplasma hyopneumoniae. Biochem Mol Biol Int 41: 821–831. 9111943 10.1080/15216549700201861

[41] Chitlaru T, Gat O, Grosfeld H, Inbar I, Gozlan Y, et al (2007) Identification of in vivo-expressed immunogenic proteins by serological proteome analysis of the Bacillus anthracis secretome. Infect Immun 75: 2841–2852 IAI.02029-06 [pii]; 10.1128/IAI.02029-06 [doi]. 17353282 PMC1932864

[42] Xolalpa W, Vallecillo AJ, Lara M, Mendoza-Hernandez G, Comini M, et al (2007) Identification of novel bacterial plasminogen-binding proteins in the human pathogen Mycobacterium tuberculosis. Proteomics 7: 3332–3341 10.1002/pmic.200600876 [doi]. 17849409

[43] Raymond BB, Tacchi JL, Jarocki VM, Minion FC, Padula MP, et al (2013) P159 from Mycoplasma hyopneumoniae binds porcine cilia and heparin and is cleaved in a manner akin to ectodomain shedding. J Proteome Res 12: 5891–5903 10.1021/pr400903s [doi]. 24195521

[44] Tacchi JL, Raymond BB, Jarocki VM, Berry IJ, Padula MP, et al (2014) Cilium adhesin P216 (MHJ_0493) is a target of ectodomain shedding and aminopeptidase activity on the surface of Mycoplasma hyopneumoniae. J Proteome Res 13: 2920–2930 10.1021/pr500087c [doi]. 24804907

[45] Kobisch M, Friis NF (1996) Swine mycoplasmoses. Rev Sci Tech 15: 1569–1605. 9190026 10.20506/rst.15.4.983

[46] Mowbray SL, Cole LB (1992) 1.7 A X-ray structure of the periplasmic ribose receptor from Escherichia coli. J Mol Biol 225: 155–175. 0022-2836(92)91033-L [pii]. 10.1016/0022-2836(92)91033-l 1583688

[47] Yu J, Kroll JS (1999) DsbA: a protein-folding catalyst contributing to bacterial virulence. Microbes Infect 1: 1221–1228. S1286-4579(99)00239-7 [pii]. 10.1016/s1286-4579(99)00239-7 10580278

[48] Jenkins C, Geary SJ, Gladd M, Djordjevic SP (2007) The Mycoplasma gallisepticum OsmC-like protein MG1142 resides on the cell surface and binds heparin. Microbiology 153: 1455–1463 153/5/1455 [pii]; 10.1099/mic.0.2006/004937-0 [doi]. 17464059

[49] Jenkins C, Samudrala R, Geary SJ, Djordjevic SP (2008) Structural and functional characterization of an organic hydroperoxide resistance protein from Mycoplasma gallisepticum. J Bacteriol 190: 2206–2216 JB.01685-07 [pii]; 10.1128/JB.01685-07 [doi]. 18192392 PMC2258871

[50] Alonso-Hearn M, Patel D, Danelishvili L, Meunier-Goddik L, Bermudez LE (2008) The Mycobacterium avium subsp. paratuberculosis MAP3464 gene encodes an oxidoreductase involved in invasion of bovine epithelial cells through the activation of host cell Cdc42. Infect Immun 76: 170–178 IAI.01913-06 [pii]; 10.1128/IAI.01913-06 [doi]. 17938223 PMC2223653

[51] Maes D, Segales J, Meyns T, Sibila M, Pieters M, et al (2008) Control of Mycoplasma hyopneumoniae infections in pigs. Vet Microbiol 126: 297–309 S0378-1135(07)00450-6 [pii]; 10.1016/j.vetmic.2007.09.008 [doi]. 17964089 PMC7130725

