# Supplementary figures and images for: Survey of Surface Proteins from the Pathogenic Mycoplasma hyopneumoniae Strain 7448 Using a Biotin Cell Surface Labeling Approach
Source: PLoS One. 2014 Nov 11;9(11):e112596. doi: 10.1371/journal.pone.0112596 (PMC4227723; doi:10.1371/journal.pone.0112596)

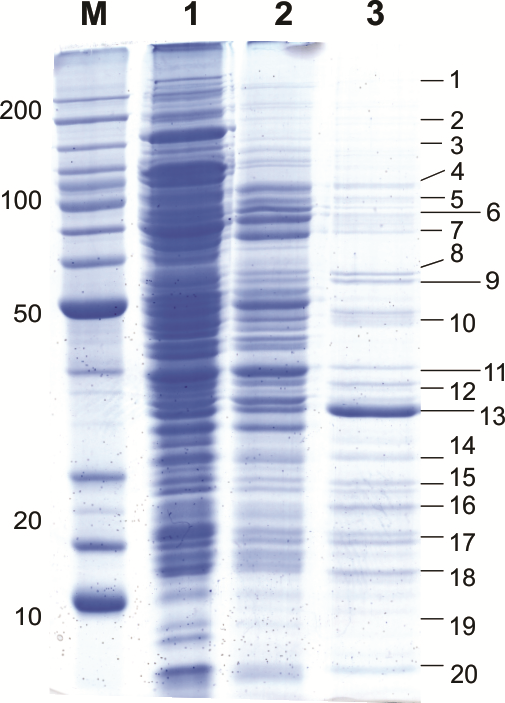

Supplement: Figure S1 — Biotin labeling and affinity capture of M. hyopneumoniae proteins from labeled lysed cell (LLC) and labeled intact cell (LIC) samples. SDS-PAGE 10%, and stained with Coomassie Brilliant Blue. Lane M – marker, Precision Plus prestained protein standards (Bio-Rad). Lane 1– crude protein extracts from M. hyopneumoniae 7448 (15 µg). Lane 2– avidin affinity capture of proteins from M. hyopneumoniae LLC samples (15 µg). Lane 3 - avidin affinity capture of proteins from M. hyopneumoniae LIC samples (15 µg). Bands 1 to 20 were subjected to Nano-LC/MS/MS analysis. (TIF) [file pone.0112596.s001.tif]
